# *In vitro* model of bacterial biofilm mineralization in complex humid environments: a proof of concept study

**DOI:** 10.3389/fbioe.2024.1496117

**Published:** 2025-01-10

**Authors:** L. Zorzetto, S. Hammer, S. Paris, C. M. Bidan

**Affiliations:** ^1^ Department of Biomaterials, Max Planck Institute of Colloids and Interfaces, Potsdam, Germany; ^2^ Department of Operative, Preventive and Paediatric Dentistry, Center of Oral Health Sciences, Charité–Universitätsmedizin Berlin, Berlin, Germany

**Keywords:** biofilm mineralization, *in vitro* model, bioreactor, microscopy, dental calculus

## Abstract

**Background:**

Bacteria in physiological environments can generate mineralizing biofilms, which are associated with diseases like periodontitis or kidney stones. Modelling complex environments presents a challenge for the study of mineralization in biofilms. Here, we developed an experimental setup which could be applied to study the fundamental principles behind biofilm mineralization on rigid substrates, using a model organism and in a tailored bioreactor that mimics a humid environment. We developed a simple yet effective method to produce rigid specimens with the desired shape.

**Materials and Methods:**

To simulate humid growth conditions, rigid specimens were conditioned with human saliva, inoculated with the chosen model bacterial strain and placed in a chamber with continuous drop-wise supply of nutritious media. The preconditioning stage did not affect significantly the bacteria proliferation, but considering this option was instrumental to future evolutions of the model, where saliva could be substituted with other substances (e.g., urine, plasma or antimicrobial solutions). Two different growth media were used: a control medium with nutritious substances and a mineralizing medium consisting in control medium supplemented with mineral precursors. Both the specimen shape and the bioreactor designs resulted from an optimization process thoroughly documented in this work. As a proof of concept, we showed that it is possible to locate the bacteria and minerals using confocal laser scanning microscopy (CLSM) and scanning electron microscopy (SEM).

**Results:**

We achieved an *in vitro* model representative of the conditions of growth and mineralization of biofilms in humid environments on a rigid substrate: something between the traditional solid-air and solid-liquid interface models.

**Conclusion:**

Such model will be useful to understand fundamental mechanisms happening in complex environments.

## Introduction

Biofilms are living biological materials formed by bacteria. These microbial tissues arise from the need for organisms to survive in challenging environments. An example of a complex challenging milieu is the mouth, where the oral microflora forms a biofilm called dental plaque. Teeth exposure to saliva results in the formation of a thin film called pellicle, which promotes bacterial cells adhesion. Then bacteria proliferate and produce an extracellular matrix (ECM), in which they get embedded. At later stages, the plaque can mineralize and form dental calculus. The result is an intricate multiphase and multi-strain microbial tissue, in which the behavior and impact of the biofilm as a whole is analyzed, rather than the individual bacteria (Cate, 2006). Oral biofilms are associated with some of the most common oral diseases, such as caries and periodontitis (Akcali and Lang, 2018).

*In vivo* and *in vitro* models combined with clinical observation led to a joint description of dental plaque maturation and mineralization, seen to occur in three phases (Jin and Yip, 2002): 1) formation of a protein layer on the tooth surface (the pellicle); 2) adhesion of early colonizer bacteria strains; 3) maturation and mineralization of the resulting biofilm. In contrast to teeth, which have a defined native but complex and/or varying composition and texture, dental restorations are made of artificial materials, which chemical composition, microstructure and surface texture are optimized by manufacturers and dentists to guarantee further proper and durable function of the repaired tooth. In this context, understanding the interactions between biofilms and dental restoration surfaces is essential to understand dental plaque and calculus formation and to design restoration materials able to tune marginal gap mineralization, which could prevent the formation of secondary caries (Jefferies, Fuller, and Boston, 2015). To date, scientific studies of the effects of surface texture and materials composition on biofilm formation in the context of dental restoration have yielded differing results. *In vitro* work showed that the choice of restoration material is more determining than surface roughness for an early colonizer bacteria strain (e.g., Streptococci) (Aykent et al., 2010); whereas *in vivo* work concluded that a smooth surface is key to impair bacterial colonization (Busscher et al., 2010). Further research thus requires elaborating strategies combining the control of *in vitro* studies and the complexity of the oral environment to identify the critical determinants in dental calculus formation.

Abiotic samples mimicking kidney stones (also known as phantom stones and artificial calculi) have been used as model to test the effectiveness of lithotripsy despite scarce availability of calculi retrieved by patients and their intrinsic variability (Nyame et al., 2015). To characterize the influence of various substances on stone growth, kidney stone farms were developed where stone fragments from patients were put in a continuous flow system of urine-type media. The growth rate of the calculi could accelerate or slow down depending on different macromolecules (e.g., phytate decrease of about 50% at sub-µM concentration). These *in vitro* models created a bridge between abiotic systems completely relying on spontaneous mineral precipitation and the *in vivo* models, where it is difficult to control or measure all the parameters (Kavanagh and Nagaraj Rao, 2007). However, when a medium close to whole urine was used, there was hardly any significant stone enlargement. This could be due to the hypothesized contribution of bacteria in mineral accumulation in the calculi (Schwaderer and Wolfe, 2017).

The traditional (top-down) approach starts from the observation of an intricate system that is simplified by gradually decreasing its complexity. In contrast, a bottom-up approach begins with the simplest model possible that can replicate the basic process interesting for the clinicians, and the complexity is gradually increased to gain in physiological relevance. In the case of dental calculus, a multi-bacterial approach is not necessary to investigate the role of phosphoproteins, as long as there is a model strain that can be used to evaluate the effects of phosphoproteins (Ennever, Vogel, and Streckfuss, 1974). Recently, *E. coli* was used to this end and intracellular mineralization has been related to the interaction between phosphoproteins and collagen fibril-like behavior of dead bacteria (Yoshikuni et al., 2023). Another mechanism of calculus formation related to polyphosphate could be due to bacterial phosphatases degrading phosphoproteins for their metabolism (Jin and Yip, 2002). Indeed, the presence of alkaline phosphatase (ALP) has been related to periodontal disease (Sanikop, Patil, and Agrawal, 2012; Malhotra et al., 2010). For kidney stones, bacteria are known to contribute to struvite stones and according to more recent evidences, they may be involved in the development of calcium oxalate and calcium phosphate stones (Schwaderer and Wolfe, 2017). In particular, *E. coli* is reported to be present in up to 35% of kidney stones extracted from patients (Tavichakorntrakool et al., 2012).

**FIGURE 1:**
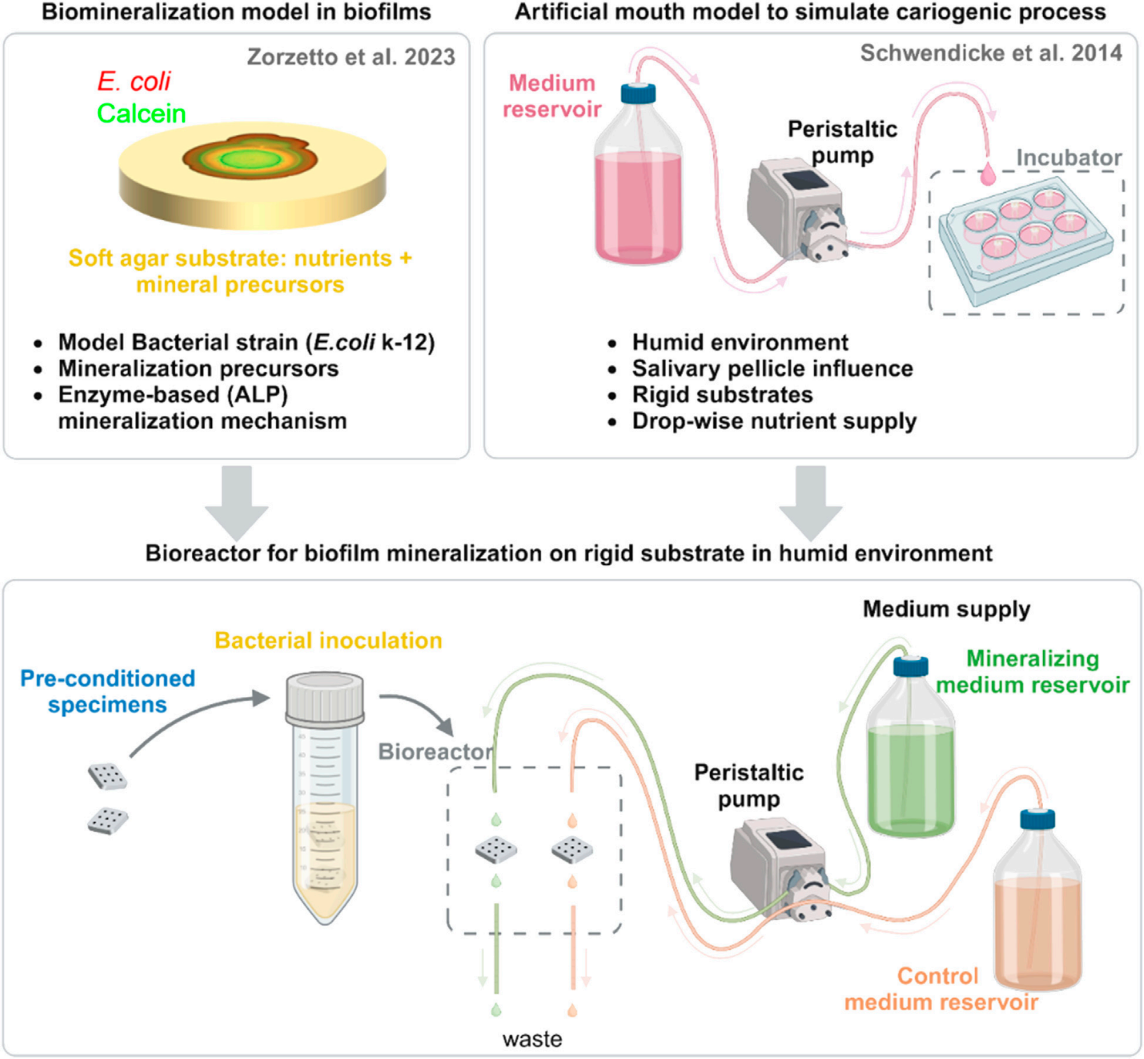
Scheme of bioreactor development and assembly. This system emerges from the combination of two previous *in vitro* models: (top left) a model used to characterize the mineralization of biofilms on soft agar with a model strain and (top right) the cariogenic artificial mouth model. From each one, we list the characteristics that were transferred to the novel bioreactor (bottom center). Here, pre-conditioned specimens are inoculated overnight in a suspension of *E. coli* K-12 strain W3110 mCherry and then put in the bioreactor chamber. Nutritious media with mineral precursors (mineralizing medium) or without them (control medium) drop on the surface of the samples. Tubes connected to a second pump operating at double the flow remove the excess liquid to avoid liquid accumulation and direct it to a waste container. Created with BioRender.com.

In a previous study, we used *E.coli* k-12 strain W3110 as a model bacterial strain to show that biomineralization at the solid-air interface can be induced by the microbial enzymatic activity of the alkaline phosphatase (ALP). Briefly, we demonstrated that *E. coli* biofilms grown on agar medium supplemented with calcium and an organic phosphate source accumulate nanocrystalline hydroxyapatite. In this process, the ALP enzyme was proposed to make the organic phosphates available to interact with the calcium to form the mineral. Indeed, when an enzyme inhibitor was added to the nutritious substrate, the mineralization was significantly delayed (Zorzetto et al., 2023). In the present work, we aim to replicate the enzymatic mineralization process in a significantly more complex environment consisting in a bioreactor that enables biofilm to grow on a rigid substrate in humid environment (Figure 1, bottom). The resulting *in vitro* model achieves mineralized biofilm colonization and growth on rigid substrate, and facilitates the analysis of the mineralized microbial tissue with confocal laser scanning microscopy (CLSM) and scanning electron microscopy (SEM). Because such model offers the possibility to test the influence of a variety of parameters (e.g., substrates, bacteria, growth conditions), it will allow the systematic study of fundamental biomineralization processes in complex physiological environments and the application of such principles to develop bioinspired mineralizing systems (e.g., geobiology and materials science).

## Results

### Bioreactor assembly

The bioreactor for biofilm mineralization adapts the design of ‘artificial mouth’ models used to simulate caries formation (Schwendicke et al., 2014) (Figure 1, bottom). Specimens made of a dental composite restoration material were first inoculated in a suspension of *E. coli* K-12 strain W3110 (Hayashi et al., 2006). This strain expresses periplasmic ALP (also known as PhoA) (Hayashi et al., 2006) and it enabled us to propose a mechanism for bacterial biofilm mineralization, which requires ALP activity (Zorzetto et al., 2023). Dental composite material was chosen both for availability and for the possibility of easily shaping and light curing. In the mouth, dental plaque grows on the surfaces or dental restorations, which are always covered with saliva. To simulate such growth conditions, dental composite specimens previously seeded with the bacteria were placed in a bioreactor chamber with continuous drop-wise supply of nutritious media for the duration of the biofilm growth and mineralization experiment from days to weeks (5 days in this proof of concept). The bioreactor chamber was designed using a computer-aided design (CAD) software and produced through an additive manufacturing technique (fused-deposition modeling) using a polymer that could withstand autoclaving (for more detailed description refer to the corresponding paragraph in material and methods). Two media were used: a control medium with nutritious substances and a mineralizing medium consisting in control medium supplemented with CaCl_2_ and β-glycerophosphate, in concentrations sufficient to lead biofilm mineralization on agar substrates (Zorzetto et al., 2023). The spontaneous precipitation of mineral at these concentrations was excluded after incubating this liquid mineralizing medium alone for 3 days and detecting no sign of aggregate formation with calcein green (Zorzetto et al., 2023). Further details of the bioreactor assembly are reported in the Materials and Methods section.

### Specimen geometry optimization

**FIGURE 2:**
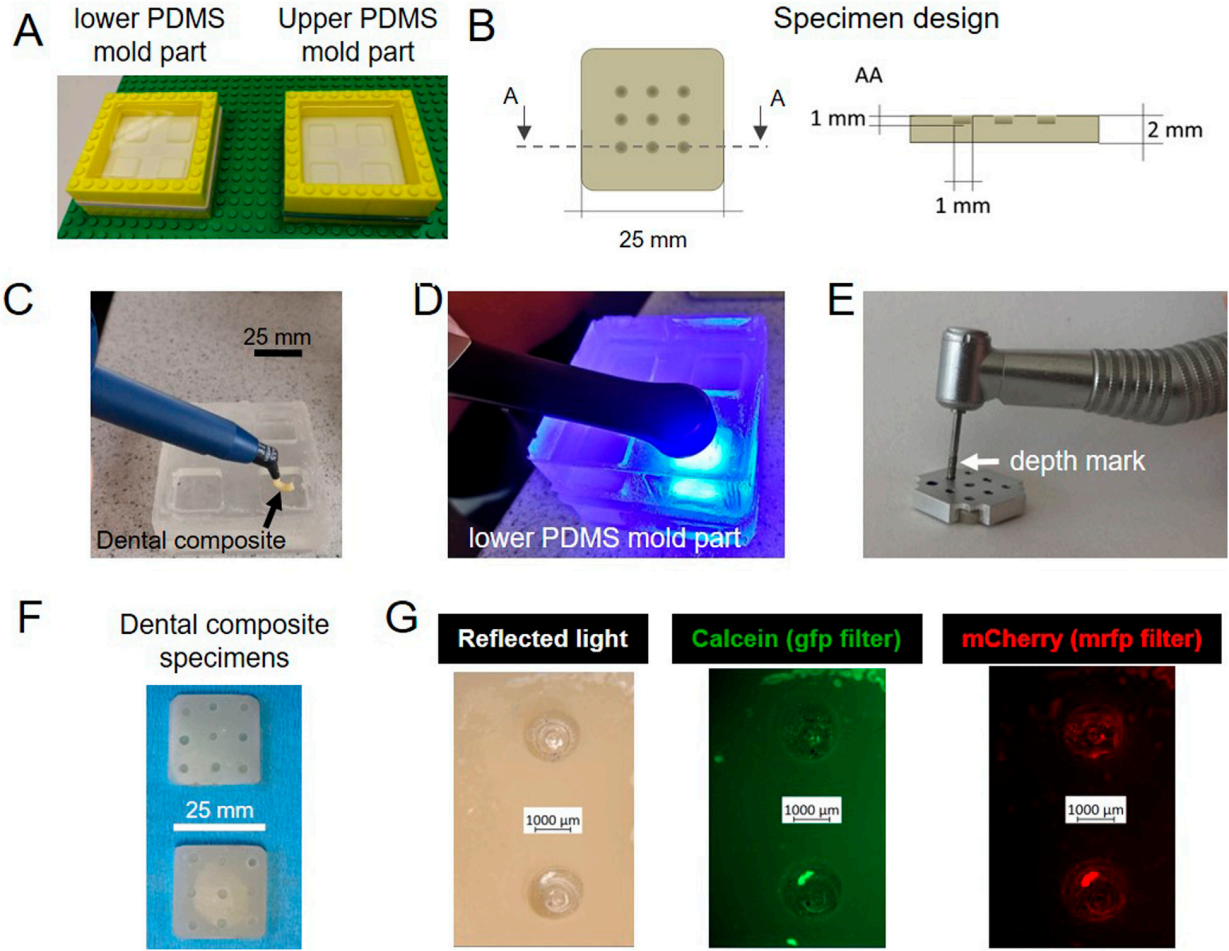
Optimized sample design. **(A)** Fabrication of the master molds in PDMS. **(B)** Sample shape and dimensions: 25 mm edge and 2 mm thickness **(C)** Dental composite paste positioned in the bottom part of the PDMS mold. **(D)** Dental paste cured with blue light. **(E)** Pits drilled in dental composite with a dental diamond drill and a metal mask to ensure the repeatability of their position and depth. **(F)** Specimens after autoclaving. **(G)** Stereomicroscopy pictures of samples incubated in the bioreactor for 5 days: biofilms and mineral tend to accumulate in the pits.

Specimens were made of commercial dental composite shaped in a polydimethylsiloxane (PDMS) mold and, among other methods possible to shape the composite, we opted for a cost-effective and easily reproducible process. The PDMS 2-side-mold was fabricated from a master mold consisting in Teflon surrounded by a LEGO™ dam (Figure 2A). The Teflon and the LEGO™ were coated with vacuum pump grease to facilitate the silicone release. For the same reason, the dam was made with modular pieces that could be easily taken apart during de-molding. The chosen specimen geometry consisted in a square plate. In the center of the square, 9 pits were drilled in a 3 × 3 pattern (Figure 2B). This design is the result of an optimization process during which we started from composites disks with different surface finishes, but these specimens did not grant a predictable biofilm distribution. We discuss further their characteristics and limitations in the Supplementary Information (Supplementary Figure S1). To obtain the desired composite specimen geometry, the uncured dental composite was placed in the bottom part of the PDMS mold and flattened with a spatula (Figure 2C). Then, the mold was closed with its upper part and pressed to even out the paste. The dental composite paste was cured with a UV lamp, shining the blue light 30 s per side (Figure 2D). The pits were then drilled in the samples with a dental drill, using a metal guide to ensure repeatability in the pit size and position (Figure 2E). The guide, which had holes in the same position as the desired pits in the specimen, was placed on top of each composite specimen and the drill tip would go through the mask holes until a pre-set mark to create corresponding pits of controlled depth in the specimen. This shape had the double function of encouraging the accumulation of liquid nutrients (and thus of bacterial tissue) in a specific region, and of avoiding evaporation during the microscopy (Figure 2F). To visualize the distribution of biofilm and mineralization on the specimens’ geometrical features, we took pictures of the samples using fluorescence stereomicroscopy after 5 days of incubation in the bioreactor. From left to right, Figure 2G shows two representative pits in reflected light (bright field), and in green and red fluorescence, which respectively report the presence of mineral (calcein green) and of bacteria (mCherry). In this case, both bacteria and mineral were present mainly in the pits, indicating a success in confining bacteria accumulation and subsequent biofilm growth to a limited region. Thanks to the versatility of these composites more complex shapes can be produced and adapted to different scenarios.

### The role of salivary pellicle

**FIGURE 3:**
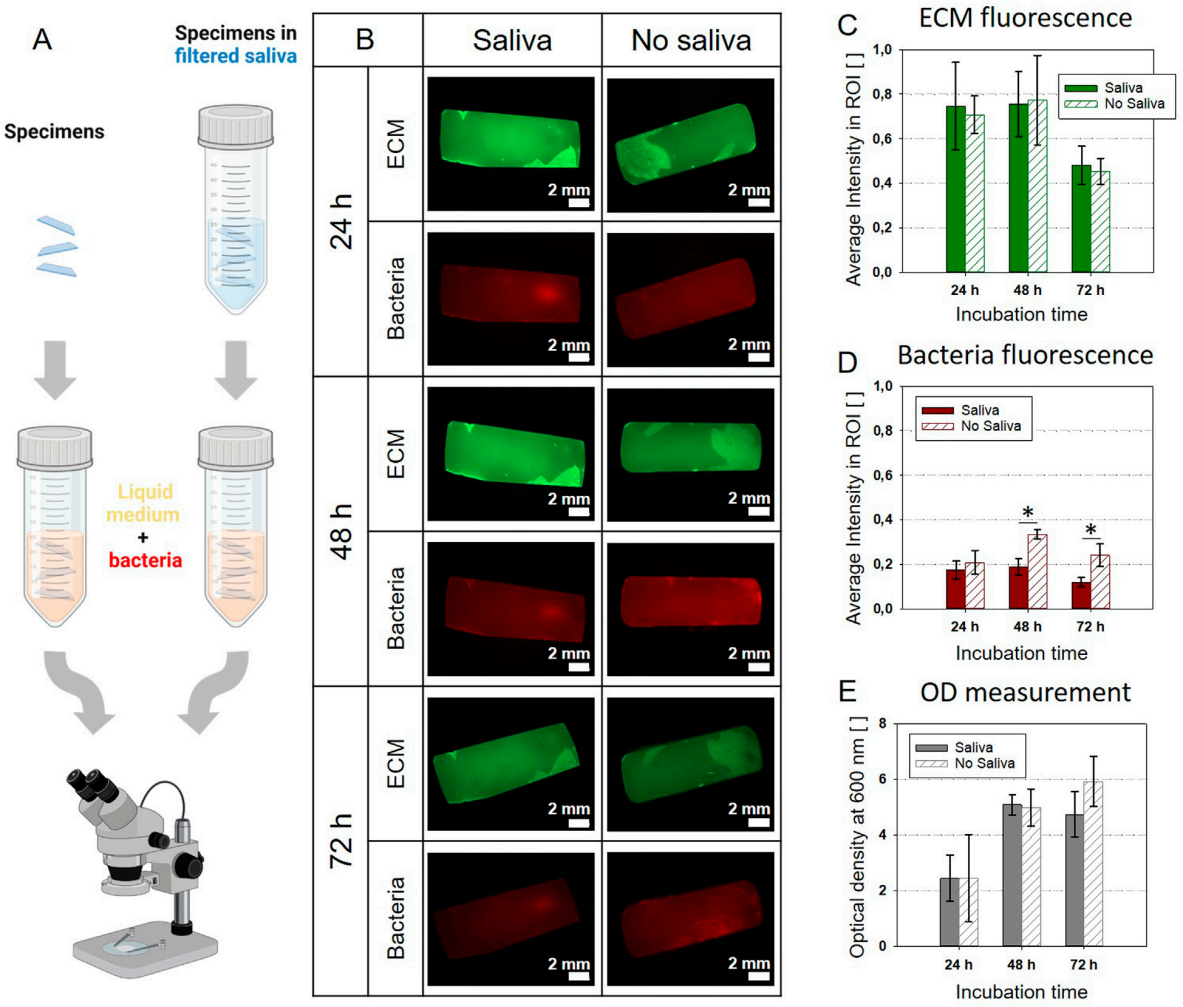
Pre-treatment with sterilized saliva: comparison between the control (no saliva) and saliva pre-treatment **(A)**. Created with BioRender.com **(B)** Stereomicroscopy pictures of specimens incubated in medium inoculated with *E. coli* mCherry with or without pre-treatment. **(C)** Average fluorescent signal of the ECM matrix shows no significant difference between samples with or without saliva pre-treatment. **(D)** Average fluorescent signal of the bacteria: after 24 h, samples without the pre-treatment show larger bacteria presence. **(E)** Optical density (OD) of the liquid cultivation medium: no significant difference between samples with or without saliva pre-treatment. The bar plots C-E report the average values and standard deviations as error bars; significant differences are indicated with asterisks (*p* < 0.01; one-way ANOVA test, n = 3).

When biofilms grow in a wet environment, the water is often loaded with proteins and biopolymers that tend to adsorb on the substrate. For example, once saliva gets in contact with dental hard tissues a thin organic film, the dental pellicle covers the surfaces. This structure, called the dental pellicle mainly consists of proteins, glycoproteins, lipids and other organic compounds (Rasputnis et al., 2021). Despite the difference in biological function and molecular composition, about 30% of salivary proteins were observed in plasma and gene ontology analysis revealed overlapping in the distribution of plasma and saliva proteomes (Yan et al., 2009). Here, we opted for a pre-treatment of the composite specimens with sterile-filtered saliva to ensure the formation of a pellicle on their surface. To test the influence of this salivary pellicle on *E. coli* biofilm growth, we performed a separate experiment where we pre-conditioned three flat rectangular composite specimens in filtered saliva before incubating them with *E. coli* W3110 mCherry in nutritious medium. The samples were autoclaved and then completely submerged in saliva in a falcon. The dental composite did not undergo macroscopically visible changes after sterilization apart from the formation of superficial stains probably related to water evaporation. The falcon was tapped to ensure the removal of potential air bubbles. The residual saliva was not washed away from the samples before incubating them in the medium. Three control samples were directly incubated in the bacteria suspension without pre-conditioning (Figure 3A). Pictures were respectively taken with the stereomicroscope 24, 48 and 72 h after starting the incubation (Lagendijk et al., 2010; Zorzetto et al., 2023). The fluorescent bacteria were used to localize their presence; whereas thioflavin S was added to the medium to mark the production of extracellular matrix (ECM). Figures 3B, C show that this step did not lead to a significant change in ECM production, but it also did not interfere with the fluorescent dyes (Supplementary Figure S2). The decline in brightness at 72 h could be due to the bleaching of the fluorescent dye, because of the repeated pictures taken on each same sample. On the other hand, the bacteria signal tends to decline for the pre-treated samples compared to the non-treated samples (Figure 3D).

This could be related to the antibacterial properties of enzymes potentially present in the saliva (Hannig, Hannig, and Attin, 2005). Yet, this effect is not visible in the values of the optical density (OD600) of the culture medium (Figure 3E). Overall, the pellicle does not appear to affect dramatically the growth of *E. coli*, but it could be fundamental for further developments of the system employing oral bacteria.

### Sample characterization

To make this *in vitro* model for bacterial biofilm mineralization on rigid substrate amenable to screening approaches, it is essential to facilitate compositional and structural characterizations of the resulting samples. We thus established the possibilities to image the biofilms with two complementary imaging techniques: confocal laser scanning microscopy (CLSM) and scanning electron microscopy (SEM).

**FIGURE 4:**
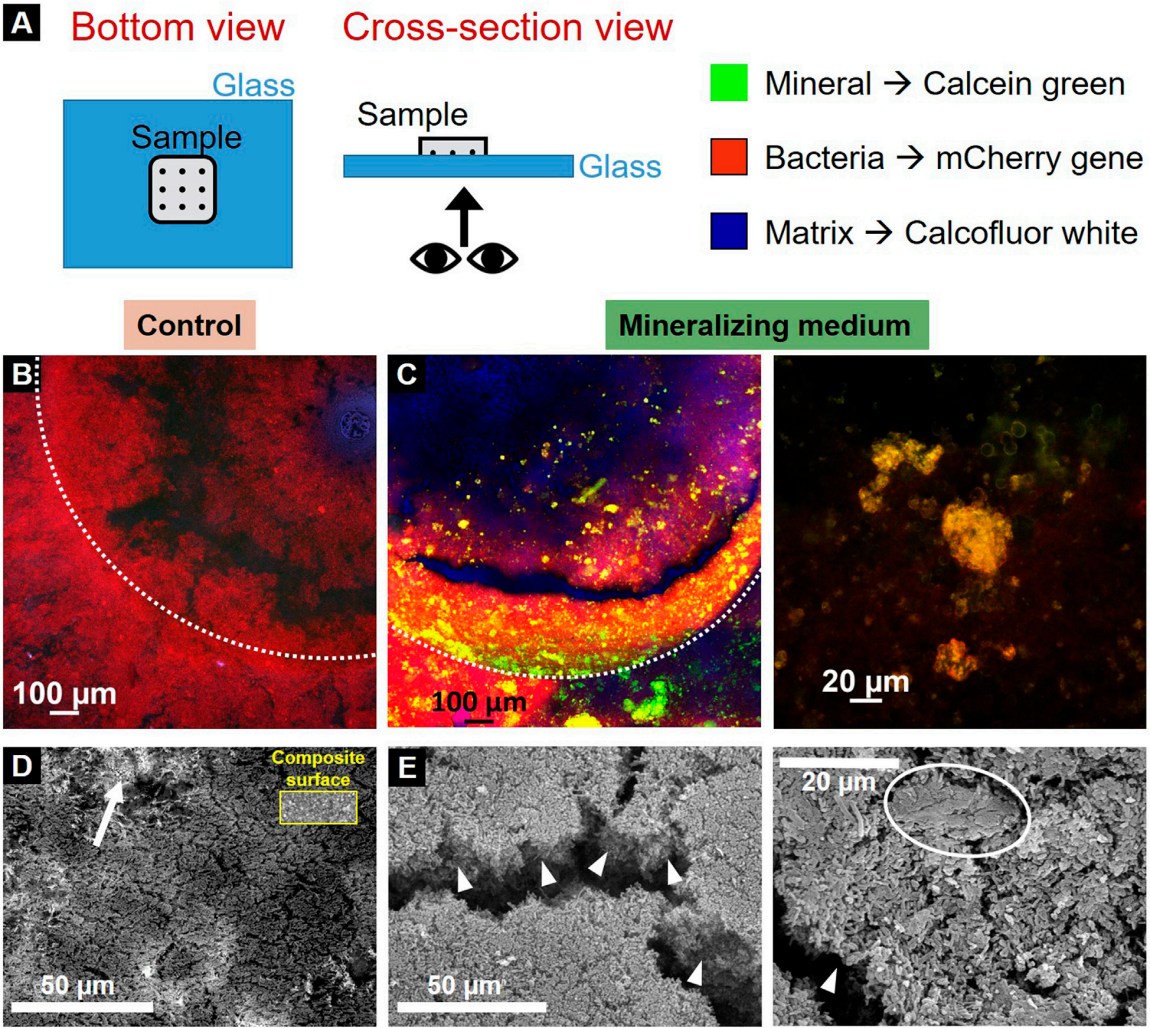
Characterization of the resulting biofilms. **(A)** Scheme of sample visualization with CLSM. Control **(B)** and mineralized **(C)** sample under confocal microscopy (dotted lines delimit the pits edges). Red channel represents the bacteria, green channel the mineral and blue channel the extracellular matrix produced by the bacteria. SEM (Secondary electron) images of the control **(D)** and mineralized samples **(E)**. An arrow shows the composite material of the specimen visible under the control biofilm for comparison the inset shows a region of bare composite (at the same magnification), arrowheads highlight the mineralized biofilm thickness and the circle indicates a mineral aggregate in the mineralized biofilm.

CLSM first provided an overview of the bacterial tissue with its different phases: bacteria, extracellular matrix and mineral. To identify them, we exploited the *E. coli* fluorescent strain producing mCherry fluorescent proteins in their cytoplasm and two fluorescent dyes provided in the liquid nutritious medium: calcein green and calcofluor white for the mineral and the ECM, respectively. In this case, calcofluor white was substituted to thioflavinS, aiming to avoid spectral overlapping with calcein green. Controls were made to check the possible crosstalk between the fluorescent dyes (Supplementary Figure S3). Samples were put upside-down on a glass coverslip and imaged without any treatment, to avoid possible artifacts from drying or fixation (Figure 4A). All the samples showed the presence of bacteria and extracellular matrix, but only samples grown in the mineralizing medium showed minerals (Figures 4B, C). Such results are in accordance with the ones obtained during sample geometry optimization, where we could show that the mineralizing medium leads to bacteria-induced mineral accumulation on the sample surface (Supplementary Figure S1).

SEM was then used to visualize the biofilms at the bacteria length-scale. Samples were observed after fixation in 70% ethanol and subsequent dehydration cycles until 100% ethanol. They underwent critical point drying (5 washing cycles) and gold coating. The pictures show the topology of the bottom of the sample pits (secondary electrons). The samples incubated in the control medium showed a mat of agglomerated bacteria forming a thin layer: indeed, the composite material on top which the biofilm grows was still visible under the bacteria (Figure 4D, arrow). For a direct comparison, the inset shows a region of bare dental composite. In contrast, the biofilms cultivated in mineralizing medium formed much thicker clusters of mineralized bacterial biofilm than their counterparts (Figure 4E, arrowheads). Additionally, there were also conglomerates of about 20 µm that hint towards mineralization (Figure 4E, circle).

## Discussion and conclusion

We established an *in vitro* model aiming at mimicking the conditions of growth and mineralization of biofilms in complex humid environments on rigid substrates: our approach is between the solid-air and solid-liquid interface models traditionally used in microbiological biofilm studies (McBain, 2009).

**FIGURE 5:**
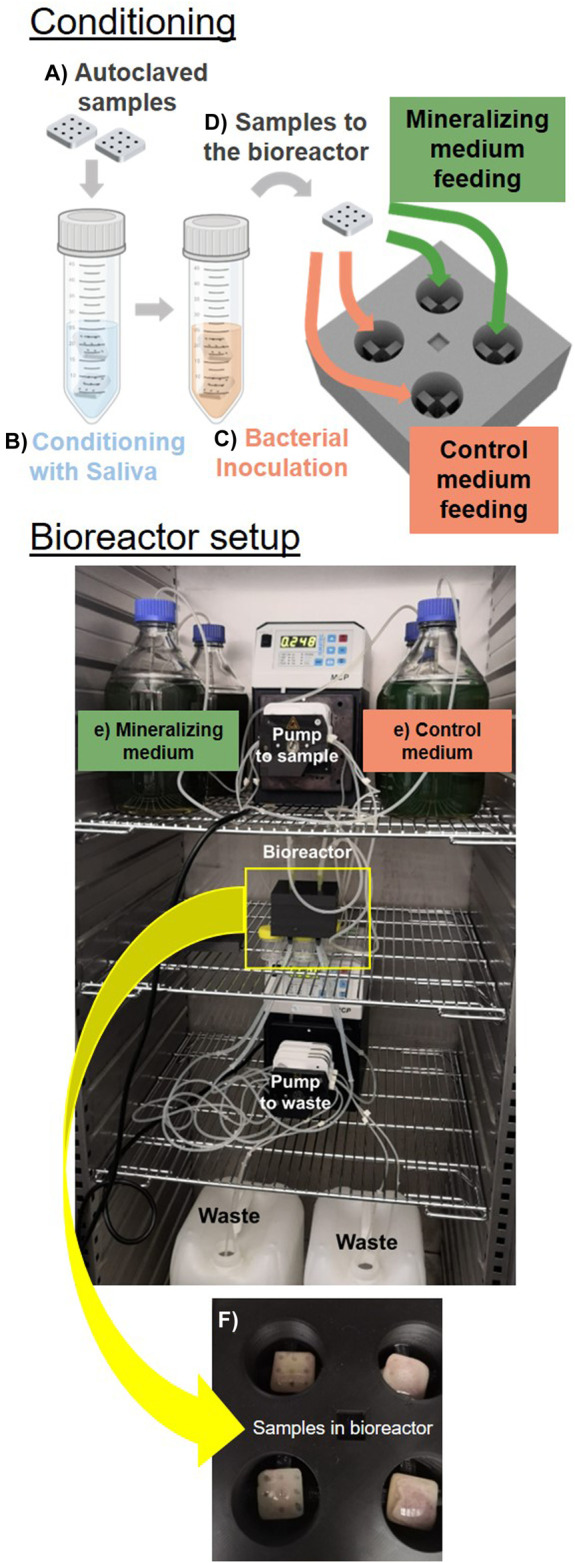
Established *in vitro* model. The assembly of the bioreactor starts from sterilized samples produced in the optimized geometry *(A)* pre-conditioned in saliva *(B)*. The samples are then inoculated with bacteria *(C)* and transfered in the bioreactor chamber *(D)*. Here bacteria are supplied with medium drop-wise *(E)*. We show samples in the bioreactor chamber after 5 days of cultivation *(F)*.

The assembly stages of the bioreactor and biofilm development can be summarized as follows (Figure 5): a) Dental composite specimens are produced according to the established geometry and sterilized. b) The specimens are pre-conditioned in sterile saliva to achieve pellicle formation and to support colonization. c) The specimens are inoculated with the bacterial strain of interest without being washed from the saliva residues. d) The samples are transferred into the bioreactor chambers. e) Sterile nutrient-rich liquid media are supplied drop-by-drop to the sample using a peristaltic pump and sterile tubing, while excess liquid is removed and sent to the waste containers by a second peristaltic pump. The flows of the two pumps are chosen to leave the surface of the samples wet, but not submerged under liquid. For this, the nutrients are provided continuously drop-wise and the pump collecting the waste is set at double the flow-rate compared to the one providing the nutrients. f) Biofilms preferentially grow in intricate regions parts of the specimen due to the accumulation and stagnation of nutrient-rich medium. g) Sample characterization demonstrates biofilm growth (and mineralization) on the composite specimens. For a more detailed description of the bioreactor assembly, the material and method section provides a comprehensive instructions to potentially reproduce the experiment.

In the present work, we used the well characterized *E. coli* W3110 as a model organism for its proven ability to both produce biofilms and induced mineralization in the presence of calcium ions and organic phosphate (Hayashi et al., 2006; Zorzetto et al., 2023). It thus appeared as an ideal candidate to establish a proof of principle and to optimize the *in vitro* model for biofilm mineralization described here. To analyze more specific problems and conditions such as dental calculus formation, our *in vitro* model might be adapted with oral bacteria: either single strains, chosen consortia, or even non-sterile saliva samples collected from donors, aiming for a model closer to the microcosm strategy. While the different stages of the workflow were tuned to create an artificial mouth model serving dental calculus research, the system is highly versatile and could be adapted to address multiple questions related to biofilm mineralization. On the other hand, this strain would be suitable for the modelling of kidney stones.

Firstly, the specimen geometry was optimized to satisfy two criteria, which are i) the facilitation of local biofilm formation and subsequent mineralization and ii) the minimal manipulation of the samples for further characterization analyses at different scales. Considering that topography is known to influence bacterial adhesion and biofilm formation at microscopic scales (Lee et al., 2021), the macroscopic geometry and surface topography of the specimens could also be modified to investigate how they influence biofilm growth and mineralization.

Secondly, we implemented the preconditioning of the composite specimen in filtered saliva to obtain an acquired pellicle as it has been used in established mouth models, when working with typical oral bacteria strains (Schwendicke et al., 2014; Sterzenbach et al., 2020). Before including this step in the bioreactor assembly, we verified the influence of the specimen preconditioning on *E. coli* biofilm growth. On the one hand, the pellicle provides binding sites such as glycoproteins for early colonizing bacteria. On the other side, saliva is rich in antimicrobial compounds, which could interfere with bacteria proliferation (Vila et al., 2019). As shown in Figure 3, saliva preconditioning does not hinder or promotes the proliferation of *E. coli* biofilm in a relevant way. However, the preconditioning could be modified using any other solution than saliva to deposit other types of coatings on the substrate (e.g., antimicrobial coating) (Ramburrun et al., 2021). Alternatively, it would be possible to combine pre-treatment and inoculation by using non-sterile saliva to inoculate the specimen with native oral bacteria. The adaptations needed to use a consortium or oral native bacteria in order to further increase the complexity of the system are multifold. In the case of the consortia, it would be necessary to choose strains that can survive symbiotically. To reach this goal, it could be required to fine tune the nutrient medium (e.g., addition of fermentable sugars), pH and the oxygen conditions. For the use of native oral bacteria, additionally to the aforementioned parameters, inherent biological variability should be taken into consideration. The configuration of the set-up with 4 separate chambers (which can be potentially increased) allows to test for different growth conditions (e.g., medium) from the same inoculum, therefore reducing considerably the variability. The possibility of increasing the number of independent chambers would be also useful to have a higher number of samples for a more accurate statistical approach.

In addition to the parameters related to surface pre-conditioning, biofilm growth parameters can also be controlled and varied on-demand. As such, the *in vitro* model will enable systematic studies to explore the influence on biofilm growth and mineralization of key parameters like the composition (e.g., mineral content), the pH, and the flow of the nutritive liquid. Likewise, the growth and mineralization of different bacteria strains can be analyzed under the same environmental conditions. Another advantage of such model is related to the 3D printed bioreactor chamber: each compartment is separate to avoid cross contamination and to allow parallel growth and analysis of different groups fed with different media. Additionally, running different conditions in parallel with the same inoculum can help to account for the inherent variability known for biological systems. The simplicity of controlling parameters is a clear advantage compared with *in situ* and *in vivo* models, which can entail ethical concerns and lower throughput, by enabling systematic studies of increasingly complex growth environments: for example, different substrates materials, different surface roughness for each material, and different oral bacterial types and co-cultures in each condition. On the other hand, *in vitro* models remain a simplification of the *in vivo* situation, thereby limiting the external validity of the system.

In this study, we demonstrated that biofilms grown with the system described can be characterized easily with electronic and optical microscopy techniques (Figure 4). However, further characterization techniques can be envisaged to perform an extensive quantitative study of the structural, mechanical and chemical properties of the resulting mineralized biofilm. This can be achieved using, for example, X-ray micro-computed tomography (microCT), micro-indentation, spectroscopy and fluorescence microscopy techniques respectively. For example, micro-indentation has been already used to evaluate stiffness and plasticity as a function of water content in the nutrient substrate (Ziege et al., 2021). This technique could also be used to perform scratch tests on the composite specimens covered with biofilms to estimate their adhesion and relate these adhesive properties of the biofilms to the surface finishing of the specimens. MicroCT was exploited to locate mineralization within the biofilm thickness at the micro scale, and wide-angle X-ray scattering was used to identify the mineral phase as being hydroxyapatite (Zorzetto et al., 2023). Similarly, these techniques could be easily adapted to the bioreactor samples to locate and identify the mineral phase at the microscale and detail the composition of the *in vitro* calculus. Moreover, thanks to the suitability of the specimen shape to be analyzed by confocal microscopes, fluorescent sensors for pH and osmotic pressure could be exploited to visualize the local distribution of such properties within living biofilms (Fulaz et al., 2019; Zhang et al., 2023).

Eventually, the proposed dynamic biofilm model can find clinical relevance beyond the dentistry field (Aarabi, Heydecke, and Seedorf, 2018) or bacteria-mediated calcification phenomena (e.g., kidney stone formation) (Saw et al., 2021; Dornbier et al., 2020). On top of that, our model could be useful in different research fields, like geobiology (Bai et al., 2021) or for development of anti-fouling surfaces.

## Materials and methods

In this paragraph, we detail how the bioreactor was assembled and how the samples were tailored, produced, and pre-treated. We also describe how the samples were analyzed after incubation in the bioreactor.

### Bioreactor design and manufacturing

The bioreactor chamber was designed to host four specimens in separate compartments to minimize the risks of cross-contamination. The entire bioreactor consisted in a bottom part where the specimens were placed and a cover through which the nutrients were deposited on the specimen’s surface (Supplementary Figure S4). To achieve this, we drew it using Fusion 360 (Autodesk, United States), a CAD software that is open source for non-commercial use. After exporting the two parts, we produced them with additive manufacturing: we used a fused deposition modeling printer with a 0.4 mm brass nozzle (Prusa MK3S from Prusa Research, Czech Republic) and a commercial filament resistant at high temperature (GreenTec Pro from Extrudr, Austria). The three-dimensional model was manufactured with a 0.1 mm layer height and the internal volume of the pieces were filled at 15% using gyroid shapes. The temperature resistance was necessary because the bioreactor chamber requires autoclaving before each experimental iteration.

### Biofilm cultivation in the bioreactor

For each iteration, four specimens made from the commercial dental composite Tetric Ceram (Ivoclar Vivadent, Liechtenstein) were manufactured as illustrated in Figure 2. They were shaped in a PDMS (Sylgard 184 silicone, Dow Europe GMBH c/o Dow Silicones, Germany) mold (monomer to crosslinking agent ration of 10:1) and cured with a dental blue light lamp (Valo Cordless; ULtradent, Köln, Germany). PDMS was chosen because it can be easily cleaned and it is transparent and thus ensured the polymerization of the dental composite through blue light. After fabrication, the specimens were autoclaved and subsequently incubated in filtered saliva for 1 h at 37°C shaking at 250 rpm. Then, the specimens were separately transferred without washing in 50 mL falcon tubes filled with 5 mL of LB medium (Luria/Miller) (Roth X968) and each vial was inoculated with a different single *E. coli* microcolony. The transfer was made under sterile hood using sterilized tweezers. The *E.coli* K-12 strain W3110 was chosen as a well-characterized model strain periplasmic alkaline phosphatase (Hayashi et al., 2006). To visualize the bacterial cells using fluorescence microscopy, the bacteria were transformed with the plasmid pMP7604 (TetR; obtained from Guido V. Bloemberg, University of Zurich) that carries the gene for the fluorescent protein mCherry (Lagendijk et al., 2010).

The specimens in the inoculated LB medium were left overnight at 37°C shaking at 250 rpm. Subsequently each seeded specimen was inserted in the bottom part of the bioreactor chamber using sterilized tweezers. To assess the potential viability difference between the different colonies, we measured the optical density (OD600) of the leftover nutritious medium used for the overnight culture and it was consistently around 0.5 after a 1:10 dilution in culture medium with a maximum 10% variability. *E. coli* biofilms were cultivated at the solid-air interface on the surface of dental composite specimens in conditions of 100% humidity. The nutrients necessary for microbial tissue growth were provided by continuously dropping liquid medium on the specimens from silicone tubing passing through the top part of the bioreactor chamber. Excess medium was removed from the bottom of the chamber to a waste container. Both tubes coming in and out the chamber had been autoclaved and were connected to peristaltic pumps to allow the nutrient to flow from the reservoirs to the chamber (0.25 mL/min) and from the chamber to the waste (0.5 mL/min) for 5 days at 37°C in aerobic conditions. The double flow rate of the waste collecting pump avoids fluid accumulation in the chamber. The length of the tubes was determined beforehand, placing empty reservoir bottles, pumps, empty bioreactor and waste. This prevented to have tubes that are too short to connect the components, or too long and risking entanglement. The tube connection was tested with sterile water in the days before the actual use to ensure that there was no leakage in the various connections. Both pumps were calibrated the day before the bioreactor assembly using the procedure recommended by the manufacturer to ensure that the desired flow was achieved and maintained during the experiment. The bioreactor CAD drawings are available upon request to the corresponding author (“Artificial_mouth_4samples.f3d” file).

Salt-free LB medium (Luria/Miller) reservoirs (control medium) were prepared with 1% w/v tryptone (Roth 8952) and 0.5% w/v yeast extract (Roth 2363) dissolved in double distilled water. For the mineralizing media, calcium ions were added in the form of CaCl_2_ (Sigma Aldrich 223506). Sodium ß-glycerophosphate (Sigma Aldrich 35675) was added to the LB medium as an organic source of phosphates. CaCl_2_ and sodium ß-glycerophosphate stock solutions were sterile-filtered (0.22 µm) and added to the autoclaved salt-free LB medium to reach a final concentration of 10 mM ß-glycerophosphate and 10 mM CaCl_2_. These CaCl_2_ and ß-glycerophosphate concentrations had lead previously to mineralization in *E. coli* biofilms cultivated on LB agar (Zorzetto et al., 2023).

Fluorescent stains were added to both media in order to identify the mineral accumulations and the extracellular matrix (ECM). For the mineral, a calcein green (Merck KGaa, 102315) stock solution (1 mM in 10 mM NaOH) was supplemented to the liquid LB media to reach a final concentration of 4 µM. Note that this type of calcein reports extracellular calcium. For the ECM, a calcofluor white (Sigma-Aldrich, 18909) stock solution (calcofluor white M2R, 1 g/L and Evans blue, 0.5 g/L) was added to the liquid media reservoirs to reach a final concentration of 3 mg/L of calcofluor white M2R and 1.5 mg/L Evans blue. Both stain stock solutions were sterile-filtered (0.22 µm) before adding them to the media reservoirs.

Apart from the samples used for confocal microscopy, all biofilms were fixed with 4% paraformaldehyde (PFA, Bioster Ar106) in phosphate-buffered saline (PBS, Sigma-Aldrich P4417). The samples were left in contact with the fixing solution for 2 h at 37°C at 60 rpm. The samples were rinsed with the aforementioned PBS solution to remove residual PFA. The experiment was repeated three times with two specimens per conditions, for a total of six specimens per condition.

### Salivary pellicle evaluation

After autoclaving, three composite specimens were incubated fully submerged in filtered saliva for 1 h at 37°C shaking at 100 rpm and subsequently put in contact with nutritious medium (20 mL) inoculated with a single colony of *E. coli* mCherry. As control, three additional autoclaved specimens were put directly in the inoculated medium. The medium consisted in 1% trypton, 0.5% yeast extract and Thioflavin S (Merck, T1892; 2 mg/mL in 70% ethanol), a fluorescent marker for biofilm extracellular matrix (ECM) used at a final concentration of 40 μg/mL. Both sample groups were cultivated for a total of 72 h and image at 24, 48 and 72 h of growth with an AxioZoomV.16 fluorescence stereomicroscope (Zeiss, Germany). At each time-step, optical density of the cultivated medium was measured. The fluorescence signal from thioflavin S was collected with the 38 HE green fluorescent protein filter set (excitation: 470/40 nm; beam splitter: FT 495 nm; emission 525/50 nm; exposure time: 225 ms). The fluorescence from mCherry was detected using the 63 He red fluorescent protein filter set (excitation: 572/25 nm; beam splitter: FT 590 nm; emission: 629/62 nm; exposure time: 500 ms).

For each channel, sample average intensity was estimated using custom-written MATLAB codes (Matlab 9.7.0 R2019b, MathWorks, Natick, MA). Fluorescence images were imported and a threshold was used to define a region of interest (ROI) corresponding to the composite surface using the function “im2bw”. This was possible thanks to the high contrast between the composites and the background. Average intensity was calculated per each channel as the mean of the intensity values of the gray scale of the original pictures in the ROI. The values are reported between 0 (black) and 1 (white).

### Fluorescence stereomicroscopy

To test the different specimen geometries, pictures were taken from air dried samples cultivated for 5 days in the bioreactor with an AxioZoomV.16 fluorescence microscope (Zeiss, Germany). Images were taken in reflected light mode with an exposure time of 15 ms. The fluorescence signal from Calcein Green was collected with the 38 high efficiency (HE) green fluorescent protein filter set (excitation: 470/40 nm; beam splitter: 495 nm; emission 525/50 nm). The exposure time was 225 ms. mCherry fluorescence was detected using the 63 He red fluorescent protein filter set (excitation: 572/25 nm; beam splitter: 590 nm; emission: 629/62 nm). The exposure time was 500 ms.

### Confocal microscopy

Confocal LSM700 microscope equipped with ×10, ×20 and ×50 objectives (Zeiss, Germany) was used to image untreated samples after 5 days cultivation in the bioreactor. Calcofluor white, calcein green and mCherry gene were excited using 408 nm, 488 nm and 555 nm laser lines, respectively.

### Scanning election microscopy (SEM)

Phenom Pro Desktop SEM (Thermofisher, Waltham, Massachusetts, United States) was used to image dried and gold-coated samples. Samples underwent ethanol dehydration with an increasing gradient of ethanol (70%, 80%, 90% and 100%), leaving the samples for 24 h in each ethanol solution. After the ethanol dehydration, samples were transferred in 100% acetone overnight before undergoing 5 cycles (10 min each) of critical point drying (CPD). Eventually, the dried samples were sputtered with gold. SEM parameters were: voltage intensity of 15 kV and pressure of 60 Pa.

### Statistical analysis

To evaluate the pre-treatment with saliva, we carried on the experiment in triplicates and we measured the average fluorescent signal for the extracellular matrix and the bacteria, as well as the optical density of the liquid cultivation medium for each sample, at each measuring time point (24 h, 48 h and 72 h after the first inoculation). To verify the statistical significance, we performed a one-way ANOVA test under the hypothesis of normal distribution considering the significance level equal to 1%.

## Data Availability

Data and analysis tools are available on request from the corresponding author or in the MPIKG library at biblio@mpikg.mpg.de.
