# Supplementary material for: In vitro model of bacterial biofilm mineralization in complex humid environments: a proof of concept study
Source: Front Bioeng Biotechnol. 2025 Jan 10;12:1496117. doi: 10.3389/fbioe.2024.1496117 (PMC11757236; doi:10.3389/fbioe.2024.1496117)
Supplement: Supplementary file 1 [file DataSheet1.pdf]

In vitro model of bacterial biofilm mineralization in complex humid environments: a proof of concept study

L. Zorzetto[1], S. Hammer[2], S. Paris[2], C. M. Bidan[1]

[1]Department of Biomaterials, Max Planck Institute of Colloids and Interfaces, Potsdam, Germany

[2]Department of Operative, Preventive and Paediatric Dentistry, Center of Oral Health Sciences, Charité - Universitätsmedizin Berlin

Supplementary materials

Early prototypes for dental composite samples

The first design for the composite specimen consisted in dental composite (Ivoclar Vivadent, Liechtenstein) embedded in Technovit 4071 (Kulzer, Germany). The composite disk was produced with a polydimethylsiloxane (PMDS) mold as described in the 'Specimen geometry optimization' paragraph. The goal of the embedding was to ease the polishing process. The overall sample has the shape of a truncated cone to avoid medium stagnating on top of the sample (Figure S1A, left). These specimens were cultivated in the bioreactor for 5 days according to the procedure described in 'Material and Methods' with calcein green to mark for the mineral presence and mCherry fluorescent bacteria to signal their presence. After the cultivation, the dried samples showed mineralization using fluorescent stereomicroscopy (Figure S1A, right). However, there was non-homogeneous distribution of the biofilm and it was not possible image the sample using and upside-down confocal microscope. A further design development consisted in avoiding using Technovit 4017 to have thinner samples more adapted for confocal microscopy (Figure S1B). After 5 days of cultivation, biofilm still mineralizes (green fluorescence from the calcein), but it is still not quite homogenous and the geometry of the sample did not allow for a simple visualization at the confocal.

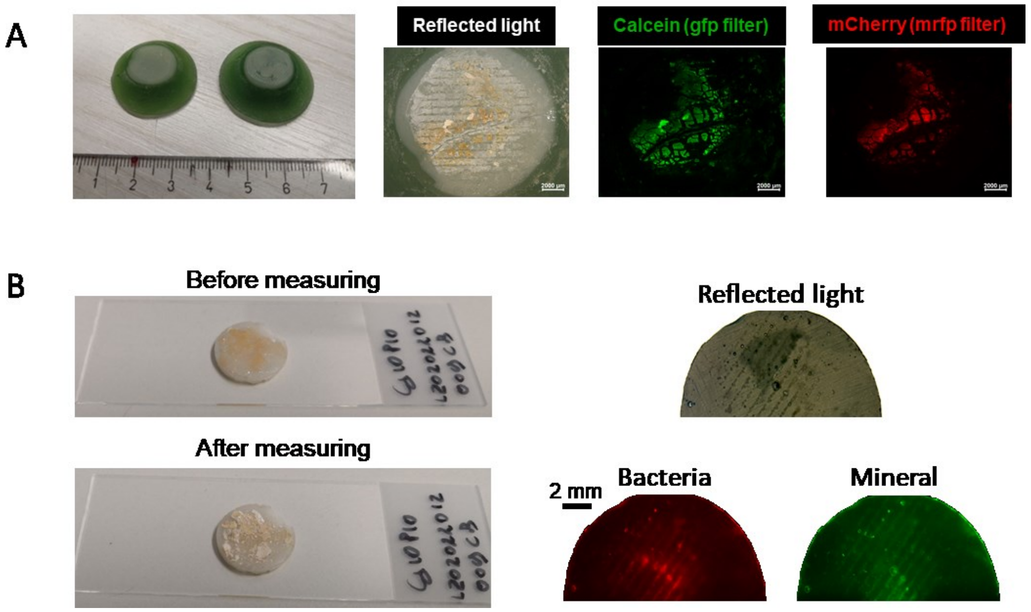

Figure S1. Preliminary composite sample designs. A) Dental composite in top of a Technovit 4071 truncated cone and fluorescent stereomicroscopy image of the dried sample. B) Dental composites disks and fluorescent stereomicroscopy image of the dried sample.

Controls for experiment on the role of salivary pellicle

Figure S2 shows dental composite samples alone, after being in contact with filtered saliva and eventually with both saliva and medium. Pictures were taken using both 38 High Efficiency (HE) green fluorescent protein filter cube (excitation: 470/40 nm; beam splitter: 495 nm; emission 525/50 nm; exposure time 225 ms) and 63 HE red fluorescent protein filter cube (excitation: 572/25 nm; beam splitter: 590 nm; emission: 629/62 nm; exposure time was 500 ms). Here, we show that neither the pre-treatment with saliva nor the nutritious medium increase the autofluorescence of the composite sample using these two fluorescent filters.

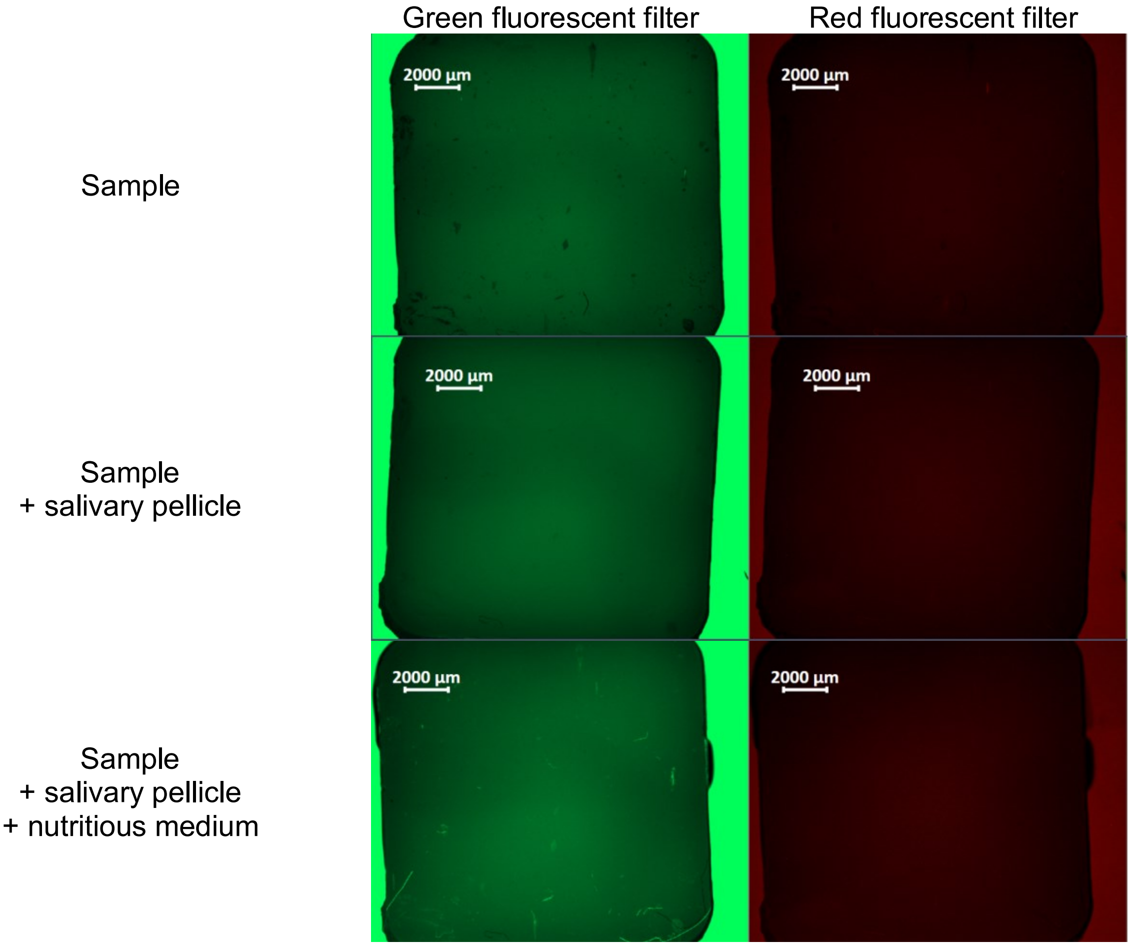

Figure S2. Controls for experiment on the role of salivary pellicle.

Controls for confocal microscopy

Figure S3 shows dental composite samples alone, after being in contact with filtered saliva, with both saliva and medium and eventually with saliva, medium and fluorescent dyes (calcofluor white and calcein). The images were obtained using a LSM700 confocal microscope at x10 magnification, with the laser wavelengths 408 nm, 488 nm and 555 nm. Similarly to the salivary pellicle controls, both the pre-treatment with saliva and the nutritious medium together with the fluorescent dyes did not present significant autofluorescence or overlap. Calcofluor white was used instead of ThioflavinS, because the

excitation/emission spectrum of ThioflavinS and Calcein Green overlap and it would not be possible to distinguish the contribution between the matrix and the mineral if used simultaneously.

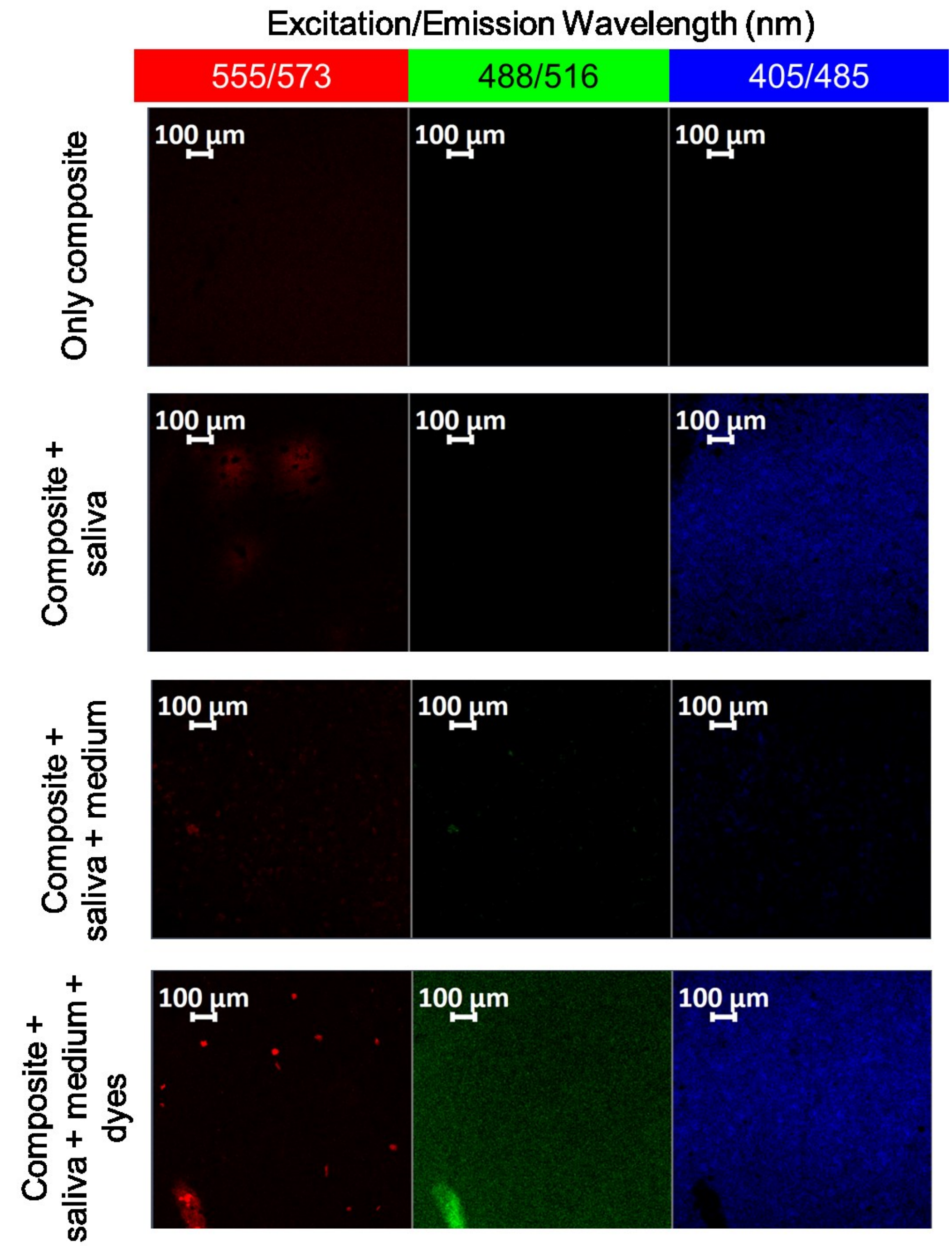

Figure S3. Controls for samples imaged with confocal microscopy.

Bioreactor Design

The computer-aid design (CAD) files of the bioreactor chamber (top and bottom) are shown in figure S4 and they are available upon request to the corresponding author as Fusion360 (Autodesk, USA) file 'Artificial_mouth_4samples.f3d'.

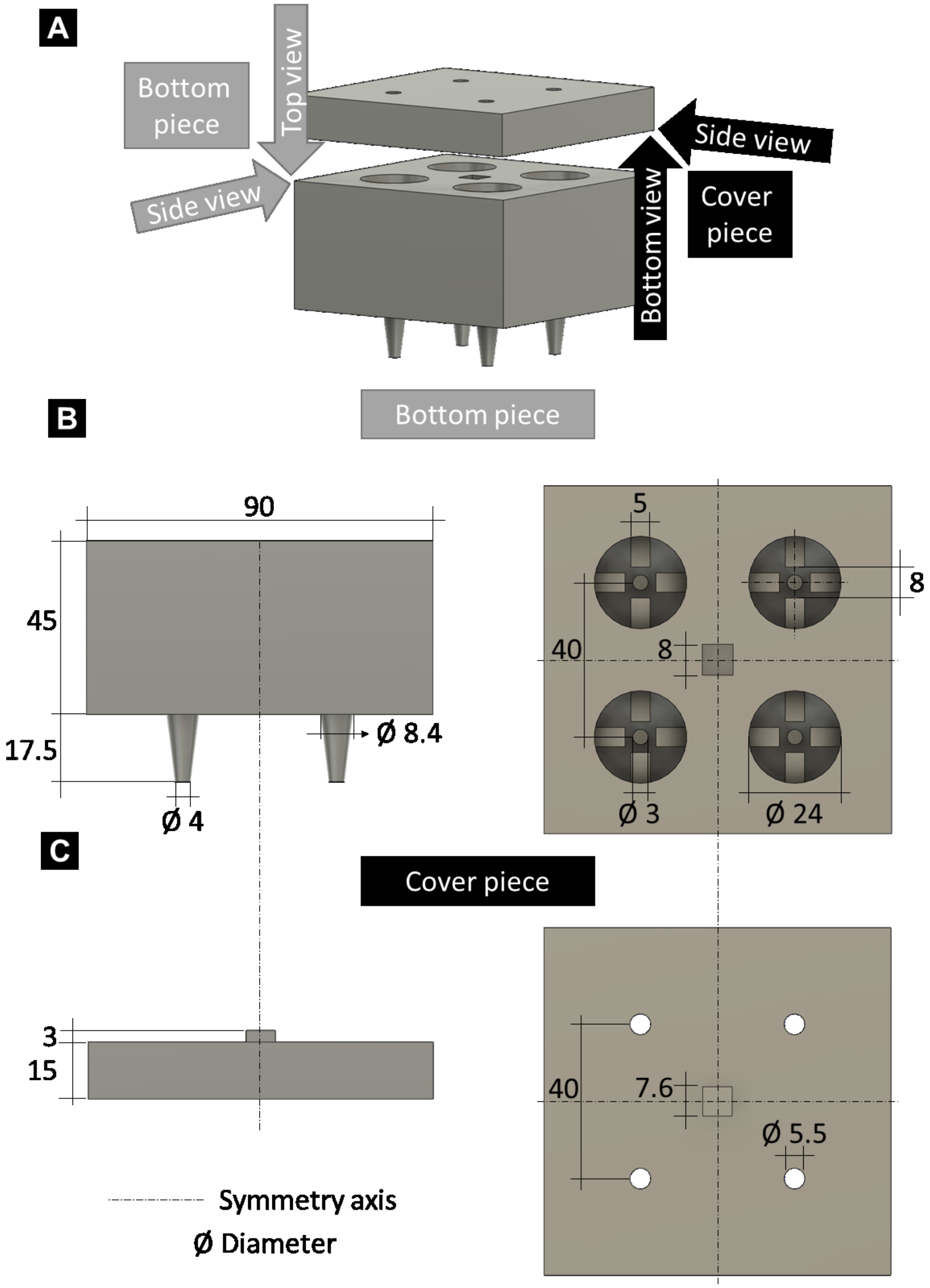

Figure S4. Bioreactor drawings with dimensions (in mm). We show a prospective view of the two parts (A) and planar views of the bottom piece (B) and the cover piece (C).
